# Clinical and Prognostic Significance of p-ANCA Positivity in Idiopathic Pulmonary Fibrosis: A Retrospective Observational Study

**DOI:** 10.3390/diagnostics13111882

**Published:** 2023-05-27

**Authors:** Alessandro Libra, Giuseppe Muscato, Giuseppe Ielo, Lucia Spicuzza, Stefano Palmucci, Evelina Fagone, Mary Fruciano, Elisa Gili, Gianluca Sambataro, Carlo Vancheri

**Affiliations:** 1 Regional Referral Centre for Rare Lung Disease, Department of Clinical and Experimental Medicine, University Hospital “Policlinico-San Marco”, University of Catania, 95123 Catania, Italy; gpp.muscato@gmail.com (G.M.); giuseppeielo@hotmail.it (G.I.); lucia.spicuzza@unict.it (L.S.); eva.fag@virgilio.it (E.F.); maryfruciano@yahoo.it (M.F.); elisagili@hotmail.com (E.G.); vancheri@unict.it (C.V.); 2 Radiology Unit I, Department of Medical Surgical Sciences and Advanced Technologies “GF Ingrassia”, University Hospital “Policlinico-San Marco”, University of Catania, 95123 Catania, Italy; spalmucci@unict.it

**Keywords:** idiopathic pulmonary fibrosis, ANCA, vasculitis, Microscopic Polyangiitis, Rheumatoid Factor, UIP pattern, UIPAF, interstitial pneumonia with autoimmune features, MPO-ANCA, multidisciplinary team

## Abstract

Perinuclear Anti Neutrophil Cytoplasmic Antibody (p-ANCA) is a serological marker of Microscopic Polyangiitis (MPA), a vasculitis associated with lung involvement potentially mimicking Idiopathic Pulmonary Fibrosis (IPF). In this study, we evaluated the role of p-ANCA in predicting clinical evolution and prognosis in a cohort of IPF patients. In this observational, retrospective, case–control study, we compared 18 patients with an IPF diagnosis and p-ANCA positivity with 36 patients with seronegative IPF, matched for age and sex. IPF patients with and without p-ANCA showed similar lung function decline during the follow-up, but IPF p-ANCA+ showed better survival. Half of IPF p-ANCA+ patients were classified as MPA for the development of renal involvement (55%) or skin signs (45%). The progression towards MPA was associated with high levels of Rheumatoid Factor (RF) at baseline. In conclusion, p-ANCA, mainly when associated with RF, could predict the evolution of Usual Interstitial Pneumonia (UIP) towards a definite vasculitis in patients, with a better prognosis compared with IPF. In this view, ANCA testing should be included in the diagnostic workup of UIP patients.

## 1. Introduction

Association between interstitial lung diseases (ILDs) and anti-neutrophil cytoplasmic antibody (ANCA) or ANCA-associated vasculitis (AAV) has been increasingly recognized during the last few years [1]. Anti-neutrophil cytoplasmic antibodies (ANCA) are autoantibodies specific for antigens located in the cytoplasmic granules of neutrophils and lysosomes of the monocytes [2]. ANCA-associated vasculitis (AAV) is a heterogeneous group of systemic vasculitides that predominantly affects small blood vessels [3]. 

Among systemic vasculitides, Microscopic Polyangiitis (MPA) is strongly associated with Perinuclear Anti Neutrophil Cytoplasmic Antibody (p-ANCA) positivity, and it is the vasculitis most frequently associated with interstitial lung disease (ILD), although patients with ILD and ANCA positivity without manifestations of systemic vasculitis have also been reported [4]. In addition, ANCA-positive conversion has been described in patients with an initial diagnosis of idiopathic pulmonary fibrosis (IPF) [5], with manifestations of systemic vasculitis in some patients [6]. The onset of ILD may precede or be concomitant with the development of a complete vasculitis syndrome in most individuals. 

Previous studies have reported that ILD precedes AAV in 14–85% of patients, it occurs concurrently in 36–67%, and it occurs after AAV in 8–21% of patients [7,8,9,10]. The age of onset of pulmonary fibrosis associated with MPA appears to be similar to that of IPF, and it is usually observed in patients aged 65 years or older, whereas the onset of MPA in patients without ILD is typically closer to 55 years. A slight male preponderance has been found in patients with ILD associated with ANCA [7,8,10,11].

The prevalence of ANCA positivity in patients with an initial presentation of interstitial pneumonia varies between 4–36% for Myeloperoxidase (MPO)-ANCA and 2–4% for Proteinase 3 (PR3)-ANCA [12,13,14,15]. In a North American retrospective study of a total of 745 patients with IPF, 25–33% of patients with an initial diagnosis of IPF with MPO-ANCA positivity developed clinical manifestations of vasculitis during a median follow-up period of 18 months [16]. In addition, studies have shown that about 10% of ANCA-negative IPF patients seroconvert during follow-up [13,14,17].

Although the 2018 IPF guidelines recommend serological testing for autoimmune diseases, they do not include an ANCA panel to rule out connective tissue disease (CTD) in patients with suspected IPF [18]. Testing for ANCA is generally not performed as part of the diagnostic workup for IPF and has not even been included in the criteria for interstitial pneumonia with autoimmune features (IPAF) because of its association with vasculitis rather than the spectrum of Connective Tissue Disease-related Interstitial Lung Diseases (CTD-ILDs) [19,20]. A 2020 international consensus on testing ANCAs beyond systemic vasculitis suggested that MPO-ANCA and PR3-ANCA should be tested in all patients with Idiopathic Interstitial Pneumonia (IIP) and included in the serologic criteria for IPAF or UIPAF, a condition of IPAF with a Usual Interstitial Pneumonia (UIP) radiological pattern even though this feature is not included among the morphological domain proposed in these criteria [19,20]. 

High-resolution computed tomography (HRCT) images are useful for detecting findings of interstitial pneumonia in the evaluation of patients with pulmonary symptoms and ANCA and/or AAV, as interstitial abnormalities may be present in a significant percentage of patients, as already shown in some studies [21,22]. Commonly described radiographic findings of AAV-ILD include ground-glass opacities, reticulation, interlobular septal thickening, consolidation, nodular pattern, and honeycombing, sometimes presenting as a UIP pattern and for that reason needing differential diagnosis with IPF. The most frequent radiological pattern in patients with MPA is UIP (up to 78% of cases), followed by nonspecific interstitial pneumonia (NSIP) (13 to 64% of cases). Considering that lung involvement is the first manifestation of disease, and above all, with a UIP pattern, it is reasonable to suppose a common misclassification of these patients as IPF, at least at the first assessment [21,23].

The aim of this study is to evaluate the possible role of p-ANCA in predicting a possible evolution towards MPA in patients previously classified as IPF while also looking for differences in prognosis between the two groups. 

## 2. Materials and Methods

### 2.1. Study Design

This was an observational, retrospective, case–control study. Data were collected from the clinical database of the Regional Referral Center for Interstitial and Rare Lung Diseases of the University of Catania, covering the period from January 2014 to December 2022. The study was approved by our local ethical committee (Ethics Committee “Catania 1”, N7819, 10 February 2022), and written informed consent to the retrospective use of the data was obtained from all patients. 

### 2.2. Inclusion Criteria

The study has the following inclusion criteria:

- Diagnosis of IPF according to available guidelines at the time of the first assessment [24] and approval of the classification after discussion in a multidisciplinary team [25];
- Detection of positivity for p-ANCA/anti-MPO using a combination of indirect immunofluorescence (IIF) of normal peripheral blood neutrophils and enzyme-linked immunosorbent assays (ELISAs);
- At least a one-year follow-up period, including lung function testing (spirometry, DLCO, and 6MWT), serological evaluation (See Section 2.5), execution of chest HRCT, and a combined pneumological and rheumatological clinical evaluation;
- The signing of written informed consent.

### 2.3. Exclusion Criteria

- Patients who do not meet inclusion criteria;
- Individuals with missing data for any variables ≥10%;
- Refusal to sign informed consent;
- Satisfaction of criteria for any CTD or systemic vasculitides at baseline.

### 2.4. Patients

**Figure 1:**
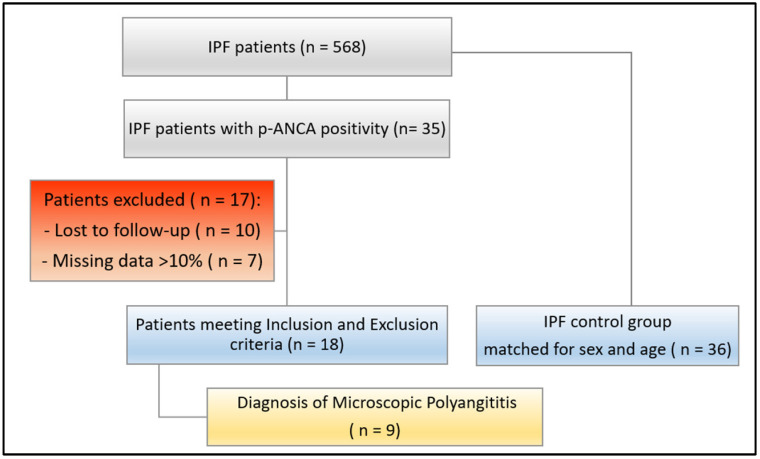
Design of the study recruitment.

We included IPF patients with positivity for p-ANCA/MPO-ANCA at the first assessment or during the follow-up as a study group. As a control group, for each IPF p-ANCA+ patient enrolled, we included 2 consecutive individuals affected by IPF without p-ANCA/MPO antibodies from our clinical database, matched for age and sex and with at least one year of follow-up. The patients’ selection is reported in Figure 1.

Patients were clinically evaluated during the observation period by a pneumologist and a rheumatologist working together with the same staff. All patients performed Pulmonary Function Tests (PFTS), including Spirometry, Diffusion Lung Capacity for Carbon Monoxide (DLCO), and 6 Minutes Walking Test (6MWT) at the baseline. Clinical or serological parameters that were noted after three months after the first visit were considered contemporary. The clinical evaluations and PFTs were made every 6 months or less, whether deemed useful in routine care. Data collected included sex, age at diagnosis, smoking status, time of first finding of p-ANCA since diagnosis of IPF, Forced Vital Capacity (FVC) obtained at the spirometry at the baseline and at 1 year, DLCO measurement at the baseline and at 1 year, and distance at 6MWT (6MWTD) at the baseline and at 1 year.

Patients were classified as MPA according to the currently proposed criteria, whereas the diagnosis of IPF was made according to the current guidelines, and vasculitis was always proven by biopsy [26]. In case of renal, cutaneous, or neurological involvement, patients were evaluated by a nephrologist, dermatologist, or neurologist.

### 2.5. Serological Assessment

P-ANCA positivity was considered whether its dosage was performed using a combination of IIF of normal peripheral blood neutrophils and ELISAs that detect ANCA specific for PR3 or MPO [27].

Patients also performed an annual, or when considered clinically necessary, serological screening for autoimmune diseases, including Rheumatoid Factor (RF), Antinuclear Antibodies (ANA), c-ANCA, p-ANCA, Extractable Nuclear Antigen (ENA) anti-cyclic citrullinated peptide (aCCP) antibodies [28]. General exams were performed at baseline and periodically during follow-up (minimum every 6 months). This panel includes complete blood count, complement fractions, transaminases, creatinine, urine tests, creatine phosphokinase, Erythrosedimentation Rate (ESR), and C Reactive protein (CRP).

### 2.6. Radiological Evaluation 

**Figure 2:**
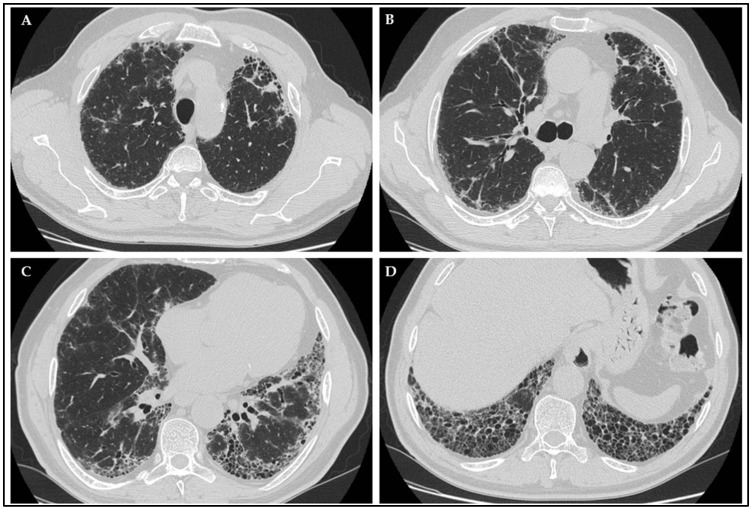
Radiological presentation of a case of IPF patient with p-ANCA positivity, showing a UIP pattern characterized by typical signs as honeycombing (**D**), traction bronchiectasis (**B**,**C**), subpleural and basal predominant distribution (**A**–**D**).

Patients performed chest HRCT, defined as computed tomography (CT) following specific protocol and slice thickness ranging between 0.625 mm and 1.25 mm, scheduled at least annually or when considered clinically necessary. Images were evaluated by a radiologist with expertise in ILD pattern definition. An example of a typical radiological UIP pattern found in the study cohort is shown in Figure 2.

### 2.7. Statistical Analysis

Statistical analysis was performed with IBM SPSS Statistics for Windows, Version 27.0. (IBM Corp, Armonk, NY, USA). We employed a Shapiro–Wilk test to evaluate data distribution. Case and control groups were compared to evaluate differences in terms of lung function decline expressed in FVC and DLCO after one year of follow-up using one-way ANOVA on ranks. We used a Chi-squared test to detect any statistical differences in the comparison of the qualitative variables between patients positive for p-ANCA whose vasculitis occurred and the individuals who did not. Finally, we performed a survival analysis using the Kaplan–Meier estimator, comparing the results of the two subgroups and between individuals with or without occurrence of vasculitis, evaluating statistical significance through Log-rank Test.

Data were presented in proportion or in mean (±standard deviation, SD), *p*-value, and 95% confidence interval (95CI). Values of *p* < 0.05 were considered statistically significant.

## 3. Results

From the analysis of our clinical database, we found 35 patients with positivity for p-ANCA detected at baseline or during the follow-up out of a group of 568 individuals with a diagnosis of IPF made at our center. From this group, we selected a total of 18 patients who met the proposed inclusion and exclusion criteria (Figure 1). We included 18 IPF p-ANCA+ patients and 36 IPF control group subjects. The study group included 12 males (66.6%) and 6 females (33.3%), with a mean age at diagnosis of 68.8 ± 7.2 years. Seven patients (38.8%) presented p-ANCA positivity at the baseline without any sign or symptom related to vasculitis, whereas the remaining patients developed p-ANCA positivity in a mean time of 32.4 ± 21.4 months. A total of 36 IPF control group patients were enrolled (24 males and 12 females, mean age 68.9 ± 7.8). All patients belonging to both groups presented a UIP pattern at chest HRCT. Characteristics of the study and control group are reported in Table 1.

Among IPF p-ANCA+ patients, six subjects were treated with pirfenidone (33.3%) and eleven with nintedanib (61.1%); one patient refused any antifibrotic drug. In the control group, 21 patients assumed treatment with pirfenidone (58.3%), and the remaining 15 subjects (41.7%) were treated with nintedanib. For both groups, there was no significant difference in terms of survival between patients treated with pirfenidone or nintedanib.

**Figure 3:**
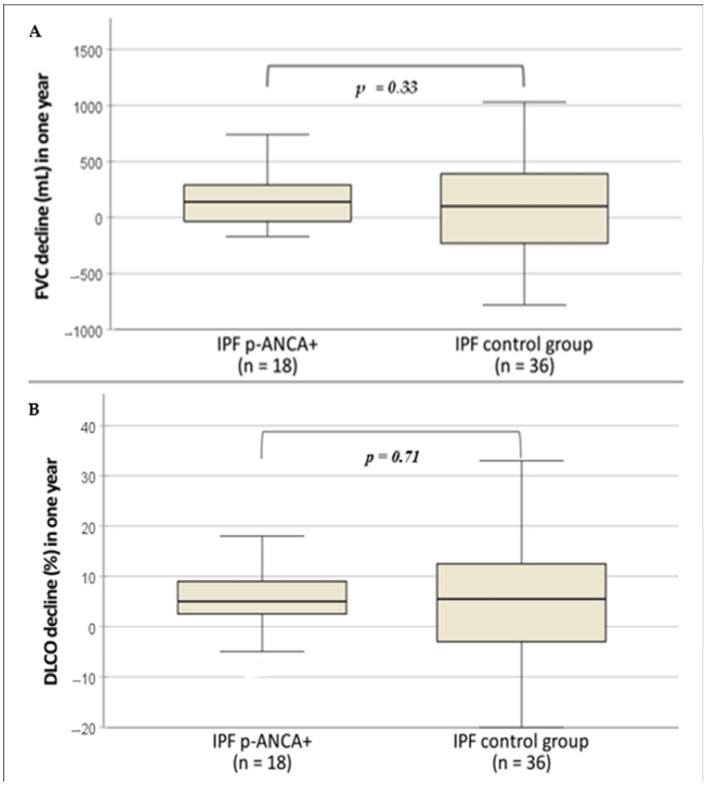
(**A**) Comparison of FVC (mL) decline after 1 year between the subgroups. (FVC: forced vital capacity). (**B**) Comparison of DLCO (%) decline after 1 year between the subgroups. (DLCO: diffusing lung capacity for carbon monoxide).

IPF patients with and without p-ANCA showed similar PFTs at the baseline and during follow-up. Complete values are reported in Table 2. The decline of FVC and DLCO is reported in Figure 3A,B. 

Among patients with p-ANCA positivity, half of them (*n* = 9, 50%) developed vasculitis during the follow-up; specifically, 5 patients (55%) had renal involvement, whereas 4 (45%) presented cutaneous manifestations. In this group, 6 patients (33.3%) died during the observation period, 2 specifically due to renal involvement, while the remaining 4 patients died due to the worsening of respiratory condition. The mean time from diagnosis to vasculitis occurrence was 29.8 ± 25.7 months. In the IPF control group, there were not any cases of vasculitis or any other systemic autoimmune disease.

With regard to the serological panel evaluated in the study group, the possible correlation between any of the items tested both at baseline and at the time of first p-ANCA detection and the occurrence of vasculitis was analyzed. Among all of these features, high levels of RF at baseline were associated with vasculitis occurrence (*p* = 0.023, X^2^ 5.14). Despite this significant result, no survival difference was demonstrated related to RF levels (Figure S1, Supplemental Materials). No associations were found to be significant among serological items dosed at concomitant detection of p-ANCA. The associations between the different items evaluated in the study are reported in Table S1. 

**Figure 4:**
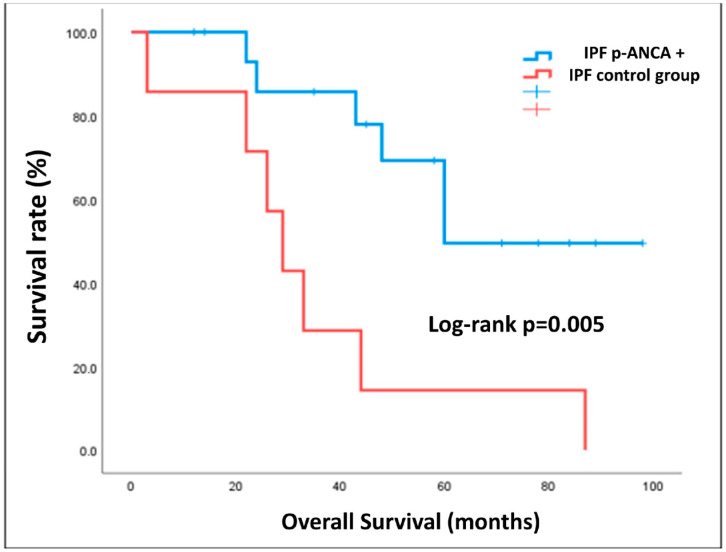
Kaplan–Meier curves of IPF positive for p-ANCA and control group.

**Figure 5:**
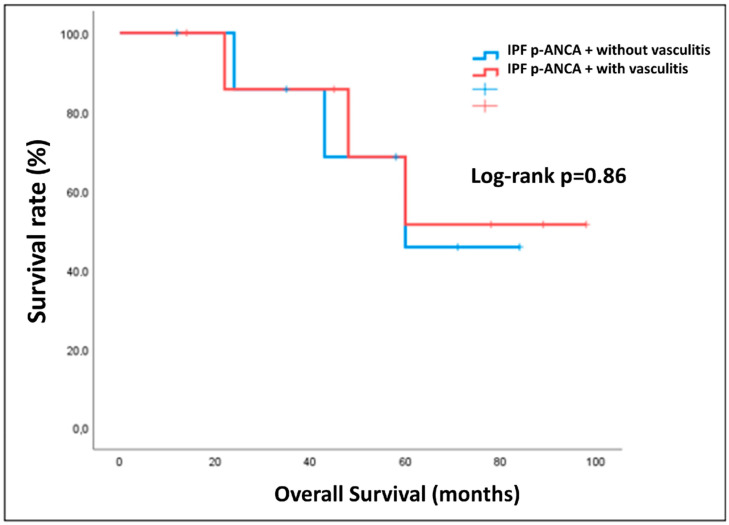
Kaplan–Meier curves of IPF positive for p-ANCA with and without occurrence of vasculitis.

Finally, the survival rate was analyzed through Kaplan–Meier estimator, demonstrating that there was a statistically significant difference (*p* = 0.005) in survival rate between IPF with positivity for p-ANCA and the control group (Figure 4). No significant differences in terms of survival were found in the subgroup positive for p-ANCA between patients with and without the occurrence of vasculitis (Figure 5).

## 4. Discussion

MPA is the systemic vasculitis with the highest prevalence of ILD in general and, in particular, with a radiological UIP pattern similar to IPF. ILD precedes the clinical onset of vasculitis in the majority of patients; therefore, a misclassification is common in these patients. Moreover, the positivity for p-ANCA could also rise during follow-up, further complicating the diagnostic assessment. Despite this, p-ANCA were not suggested either in the diagnostic workup proposed for IPF or in IPAF criteria; therefore, the diagnostic assessment of these patients is actually very difficult at their clinical onset. It should also be noted that IPAF criteria also include a “morphological domain” created by the authors in order to limit the enrollment of UIP patients [19,20]. One of the most important merits of the IPAF criteria is to include patients with an autoimmune flavour at risk of developing definite conditions. The exclusion of p-ANCA and UIP patients from these criteria probably produces the loss of a significant proportion of patients capable of actually developing vasculitis. In the manuscript, the aim to include patients with connective tissue diseases (CTDs), limiting the inclusion of vasculitides, was clearly explained. However, the recognition of an autoimmune pathway underlying an ILD could also suggest a possible benefit from treatment with immunosuppressants. In light of the already established concept that a working diagnosis of IPF should be reviewed at regular intervals as the diagnosis may change, it is necessary to re-examine patients in whom the longitudinal course of the disease is discordant with the previously established multidisciplinary diagnosis, such as in our case positive p-ANCA or new onset of clinical signs compatible with systemic vasculitis [29].

The recognition of vasculitis among UIP patients has several advantages. First of all, vasculitides are conditions associated with systemic involvement, potentially fatal (for example, when the kidney is involved). Secondarily, despite a fibrotic pattern almost identical to IPF, growing evidence suggested a potential role of immunosuppression when the UIP pattern is associated with an autoimmune disease [30,31].

In our IPF cohort, we found a p-ANCA positivity in 6.6% of patients, a proportion in line with what was reported in previous studies [13,17], but it could be underestimated, considering the absence in the usual diagnostic workup for IPF of these autoantibodies.

Seronegative patients showed a worse prognosis compared with IPF p-ANCA+ subjects. This data could support a different pathogenesis among a similar radiologic pattern, supporting the possibility that p-ANCA actually serves as a potential marker of vasculitis in these patients. We did not note any difference in mortality in IPF p-ANCA+ patients with and without the occurrence of vasculitis. This data could be biased by the limited number of patients included in the study and should be evaluated in depth. However, it should be noted that among the patients with p-ANCA positivity, 2 patients died of renal failure secondary to vasculitis, while the remaining 4 patients died of pulmonary causes. It is, therefore, reasonable to suppose that rapid identification of UIP patients secondary to MPA could allow an early pharmacological treatment that could avoid or limit lung damage, thereby improving prognosis.

Moreover, from the data we collected, the association between the onset of vasculitis and increased levels of RF at baseline was statistically significant, making it a potential prognostic laboratory marker to keep in consideration and to indicate those patients for whom a more careful clinical and laboratory follow-up aimed at early detection of systemic signs of vasculitis (e.g., physical-chemical examination of urine, renal function, etc.) is necessary.

This association could be explained considering that patients with AAV have increased production of immunoglobulin G (IgG) [32], and RF is a relatively nonspecific autoantibody against the Fc portion of IgG. RF and IgG contribute to the formation of immune complexes that contribute to the disease process. Furthermore, the activation of the complement pathway is known to play a crucial role in the pathogenesis of AAV and is implicated in the development of glomerulonephritis [33].

The presence of increased RF in vasculitis has been documented, with some controversy: in some studies, the positivity of RF in patients with AAV did not affect the relapse-free survival rate of AAV during follow-up [34], but in other studies, RF titers in patients with AAV correlated significantly with disease activity and inflammatory markers [35]. To our knowledge, this is the first study to evaluate RF in patients with an idiopathic UIP pattern on a chest CT scan and p-ANCA positivity as a possible predictive factor for the development of vasculitis.

From the therapeutic point of view, all patients enrolled in our study were treated with antifibrotics, excluding one subject who refused the assumption of any drug. The effectiveness of these drugs has been proved by several clinical trials. In some studies, the action of these molecules resulted in improvements when associated with immunosuppressants [36], although it should be noted that the treatment with immunosuppressants in IPF was also associated with an increased risk of acute exacerbation (AE) [37]; however, UIP-IPAF patients had significant benefit from the immunosuppressive treatment, despite the fact that the nuanced clinical picture did not allow for a specific classification as an autoimmune disease [30,38,39]. One of the current unmet needs for the coming years is the recognition of a subgroup of UIP patients who may have benefited from combined treatment with immunosuppressants and antifibrotics. For this reason, after the development of appropriate clinical trials, it is mandatory to modify IPAF criteria [40].

Current recommendations about the treatment of AAV do not mention how to manage patients affected by lung fibrosis [41]. On the other hand, the use of both approved antifibrotic treatments in IPF (pirfenidone and nintedanib) may help patients who have developed this complication in the context of an AAV. The INBUILD study showed the effectiveness of nintedanib on progressive fibrosing-ILD (PF-ILD) other than IPF, and nintedanib might have the potential to reduce AE occurrence [36].

Post hoc analysis of the INBUILD study suggested a treatment benefit of nintedanib in all patient subgroups with PF-ILD, including autoimmune ILD, although the study was not powered to provide evidence to address this specific claim [42]. In the RELIEF study, although the quality of the evidence was rated as low, in patients with fibrotic ILDs other than IPF who deteriorate despite conventional therapy, adding pirfenidone to existing treatment might attenuate disease progression as measured by a decline in FVC in patients with collagen or vascular diseases (i.e., CTD-ILDs), fibrotic nonspecific interstitial pneumonia, chronic hypersensitivity pneumonitis, or asbestos-induced lung fibrosis [43].

These data may encourage pilot studies and ongoing clinical trials evaluating the use of antifibrotics for patients with MPA complicated by ILD (ClinicalTrials.gov NCT03385668). 

The main limitations of the study are the retrospective design and the small number of patients included, although due to the rarity of the two diseases, which therefore needs confirmation in larger studies.

We have to consider even some of the merits of this study. This was the first study demonstrating a possible prognostic role of p-ANCA in patients with IPF/UIP radiological pattern, despite a similar clinical and functional course; moreover, results about RF could shine a light on possible prediction of vasculitis occurrence and evolution to MPA.

## 5. Conclusions

Patients with a UIP radiologic pattern on chest CT and a p-ANCA positivity need close clinical and laboratory follow-ups aimed at early detection of signs of vasculitis. RF may be a predictive marker for the development of vasculitis in patients with IPF and ANCA positivity. The inclusion of ANCA in IPAF criteria, together with the removal of the “morphological domain” (aimed to limit the inclusion of UIP patients), could improve the recognition of ILD patients at risk of developing vasculitis. More studies are needed to demonstrate the efficacy of antifibrotic treatments in patients affected by this condition.

## Figures and Tables

**Table 1:** Demographic data of the population enrolled in the study.

	IPF p-ANCA+ (*n* = 18)	IPF Control Group (*n* = 36)	*p*-Value
Mean Age (years)	68.83 ± 7.21	68.83 ± 7.76	1
Sex	6 females (33.3%) 12 males (66.6%)	12 females (33.3%) 24 males (66.6%)	1
Smoking history	5 never (27.78%) 13 former (72.22%)	15 never 19 former 2 current	0.59
Mean Smoking exposure (P-Y)	35.53 ± 32.36	43.26 ± 40.02	0.88
Vasculitis	50%	0	<0.001
Deaths	6 (33.33%)	8 (22.22%)	0.38
Mean time of Follow-up (months)	52.56 ± 26.91	50.80 ± 22.96	0.79
Mean time to death since diagnosis (months)	42.83 ± 16.86	35.25 ± 24.22	0.41

**Table 2:** Pulmonary Function Tests values of the two subgroups at the baseline and after one year.

	IPF p-ANCA+	IPF Control Group	*p*-Value
Mean FVC (mL) at the baseline	2906.67 ± 707.53	2585.83 ± 822.38	0.282
Mean FVC (%) at the baseline	90.72 ± 18.60	81.30 ± 17.92	0.127
Mean DLCO (%) at the baseline	58.40 ± 17.92	54.08 ± 16.40	0.330
Mean 6MWTD (m) at the baseline	373.75 ± 125.45	379.03 ± 129.69	0.850
Mean FVC (mL) after 1 year	2668.12 ± 863.02	2508.61 ± 814.32	0.744
Mean FVC (%) after 1 year	85.89 ± 19.06	79.52 ± 18.44	0.108
Mean DLCO (%) after 1 year	50.22 ± 18.10	50.67 ± 13.77	0.837
Mean 6MWTD (m) after 1 year	352.14 ± 142.26	367.88 ± 130.72	0.632

Legend: FVC: forced vital capacity; DLCO: diffusing lung capacity for carbon monoxide; 6MWTD: distance at the 6 min walking test.

## Data Availability

The data presented in this study are available on request from the corresponding author. The data are not publicly available due to ethical reasons.

## Supplementary Materials

The following supporting information can be downloaded at: https://www.mdpi.com/article/10.3390/diagnostics13111882/s1, Table S1: Association of serological features at the baseline with the occurrence of Vasculitis, Figure S1: Kaplan–Meier curves of subgroups defined by RF levels.
