# Supplementary material for: Clinical and Prognostic Significance of p-ANCA Positivity in Idiopathic Pulmonary Fibrosis: A Retrospective Observational Study
Source: Diagnostics (Basel). 2023 May 27;13(11):1882. doi: 10.3390/diagnostics13111882 (PMC10253038; doi:10.3390/diagnostics13111882)
Supplement: Supplementary file 1 [file diagnostics-13-01882-s001.zip › diagnostics-2401368-supplementary.pdf]

diagnostics

Table S1. Association of serological features at the baseline with occurrence of Vasculitis.

Item related to Vasculitis occurrence	*p*	95%CI
CRP	0,522	
ESR	0,453	
RF	0,023 (X^2 5,14)	1.09-10.24
ANA ≥ 1:80	0,058	
Blood eosinophils ≥ 1000	n.a.	n.a.
c-ANCA/anti-PR3	0,712	

Legend: CRP: C reactive protein; ESR: erythrosedimentation rate; RF: rheumatoid factor; ANA: anti-cytoplasmatic antibodies; c-ANCA: perinucleolar anti-neutrophil cytoplasmic antibodies.

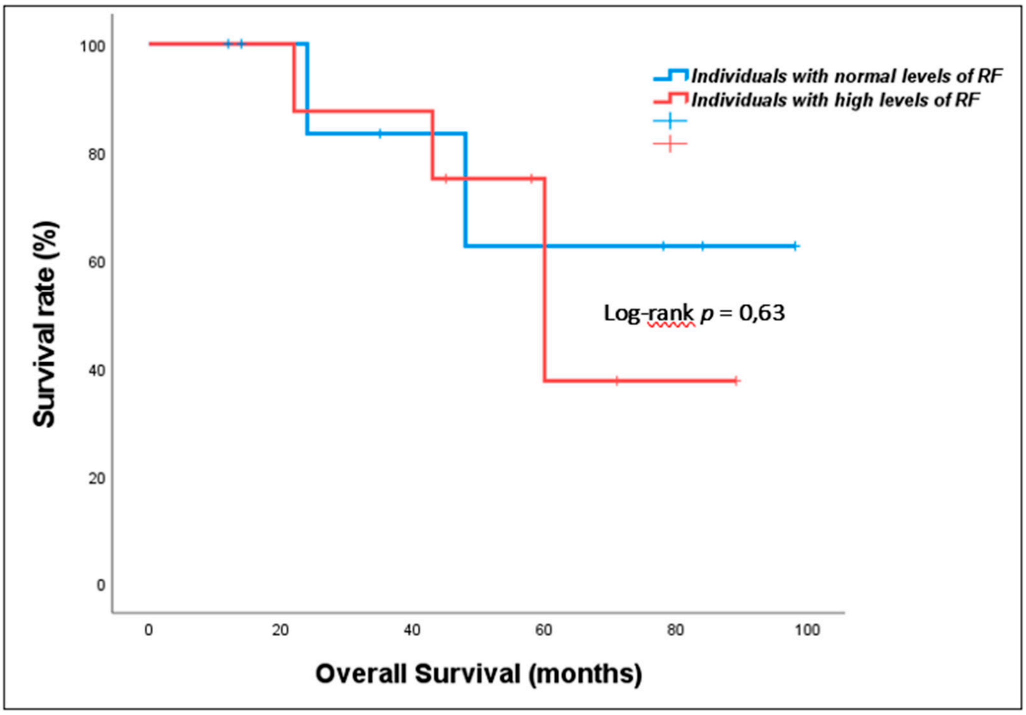

Figure S1. Kaplan-Meier curves of subgroups defined by RF levels. (RF: Rheumatoid factor).
